# Supplementary figures and images for: Single-cell multiomics data analysis of potential receptors and therapeutic drugs for epilepsy patients comorbid with depression
Source: PLoS One. 2026 Apr 22;21(4):e0347526. doi: 10.1371/journal.pone.0347526 (PMC13102190; doi:10.1371/journal.pone.0347526)

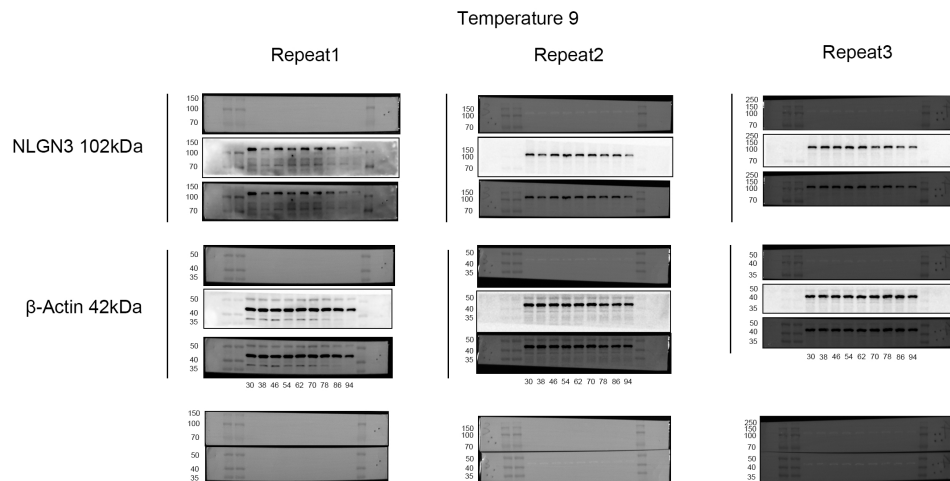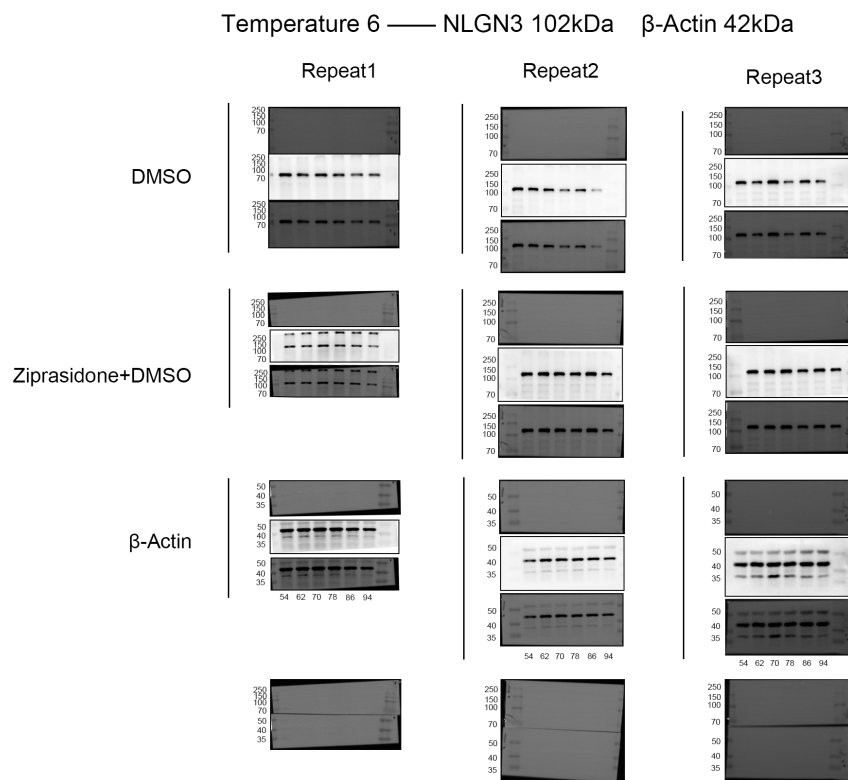

Supplement: S1 File — (PDF) [file pone.0347526.s001.pdf]
